# Comparison of Trauma Scoring Systems for Predicting Mortality in Emergency Department Patients with Traffic-Related Multiple Trauma

**DOI:** 10.3390/diagnostics15121563

**Published:** 2025-06-19

**Authors:** Murtaza Kaya, Harun Yildirim, Mehmet Toprak, Mehmed Ulu

**Affiliations:** 1 Department of Emergency Medicine, Medical Faculty, Kutahya Health Sciences University, 43020 Kutahya, Turkey; harun.yildirim@ksbu.edu.tr; 2 Emergency Department, Kutahya City Hospital, 43020 Kutahya, Turkey; mehmet.toprak@ksbu.edu.tr; 3 Department of Emergency Medicine, Adiyaman University, 02040 Adiyaman, Turkey; mehmed.ulu@ksbu.edu.tr

**Keywords:** trauma severity index, ISS, APACHE II, traffic accidents, mortality, emergency medicine, Glasgow Coma Scale

## Abstract

**Background/Objectives:** Trauma scoring systems are essential tools for predicting clinical outcomes in patients with multiple injuries. This study aimed to compare the performance of various anatomical and physiological scoring systems in predicting mortality among patients admitted to the emergency department following traffic accidents. **Methods:** In this prospective observational study, trauma patients presenting with traffic-related injuries were evaluated using seven scoring systems: ISS, NISS, AIS, GCS, RTS, TRISS, and APACHE II. Demographic data, clinical findings, and laboratory values were recorded. The prognostic performance of each score was assessed using ROC curve analysis, and diagnostic metrics including sensitivity, specificity, and likelihood ratios were calculated. **Results:** Among 554 patients included in the study, the overall mortality rate was 2%. The TRISS and GCS scores demonstrated the highest predictive performance, each with an AUC of 0.98, sensitivity of 100%, and specificity exceeding 93%. APACHE II followed closely with an AUC of 0.97, also achieving 100% sensitivity. NISS (AUC = 0.92) and ISS (AUC = 0.91) were effective anatomical scores, while RTS showed moderate predictive value (AUC = 0.90). Strong correlations were noted between ISS, NISS, and AIS (Rho > 0.85), while RTS was negatively correlated with these anatomical scores. All scoring systems showed statistically significant associations with mortality. **Conclusions:** TRISS, GCS, and APACHE II were the most effective trauma scoring systems in predicting mortality among emergency department patients. While complex models offer higher accuracy, simpler scores such as RTS and GCS remain valuable for rapid triage. The integration of both anatomical and physiological parameters may enhance early risk stratification and support timely decision-making in trauma care.

## 1. Introduction

Multiple trauma is defined as injuries involving at least two of the following regions: the head and neck, thorax, abdomen, or extremities. Alternatively, it can be defined as fractures of at least two long bones [1]. Various scoring systems, such as the Abbreviated Injury Score (AIS), New Injury Severity Score (NISS), Injury Severity Score (ISS), Glasgow Coma Scale (GCS), and Trauma and Injury Severity Score (TRISS) are utilized to assess the severity of injuries in trauma patients [2,3]. Each scoring system has specific strengths and limitations. For instance, the ISS is calculated by summing the squares of the highest AIS values from the three most severely injured body regions. However, since it considers only one injury per region, it may underestimate the total injury burden. Moreover, identical ISSs from different anatomical regions may not reflect the same mortality risk, reducing its predictive precision [4].

The Revised Trauma Score (RTS) is a physiological scoring system that utilizes easily measurable parameters such as the Glasgow Coma Scale (GCS), systolic blood pressure, and respiratory rate, enabling the rapid assessment of trauma patients, particularly in emergency departments. Its advantage lies in its quick applicability, aiding in the prioritization of critically injured patients. However, compared to anatomical scoring systems, the RTS may have limitations in predicting long-term outcomes [5].

Comparative studies on thoracic trauma patients have shown that both ISS and NISS effectively predict mortality, with NISS offering greater sensitivity in more severe injuries [4]. Likewise, the APACHE II score has demonstrated a significant association with mortality among trauma patients admitted to intensive care units, underlining its value as a prognostic tool in critical care settings [6].

Numerous studies have investigated the association between trauma scoring systems and mortality, most of which have been conducted in intensive care units or specialized trauma centers [7,8]. More recently, studies have begun to focus on trauma scoring systems in emergency department settings, reflecting interest in their use in real-world, early-phase trauma care [9,10].

In this research, we calculated the ISS, NISS, AIS, GCS, RTS, TRISS, and APACHE II scores for patients with multiple trauma admitted to the emergency department following traffic accidents. We evaluated the effectiveness of these scoring systems in predicting mortality.

## 2. Materials and Methods

### 2.1. Study Design and Setting

This prospective cross-sectional study was conducted between 1 April 2022, and 1 April 2023, in the emergency department of a tertiary university hospital with an annual patient volume of approximately 250,000 visits. The department is organized into designated care zones based on triage acuity: green (low acuity), yellow (moderate acuity), red (high acuity), and a dedicated critical care area. Each shift includes 1–2 emergency medicine residents in the green zone, 2 in the yellow, 2 in the red, and one emergency medicine specialist overseeing the critical care area.

Patients presenting with multiple trauma are initially evaluated by an emergency medicine specialist. When emergency surgical intervention is indicated, relevant surgical departments—such as general surgery, neurosurgery, or orthopedics—are promptly consulted. Critically ill trauma patients are treated in the resuscitation area under the direct supervision of the emergency medicine specialist.

### 2.2. Participants

**Figure 1:**
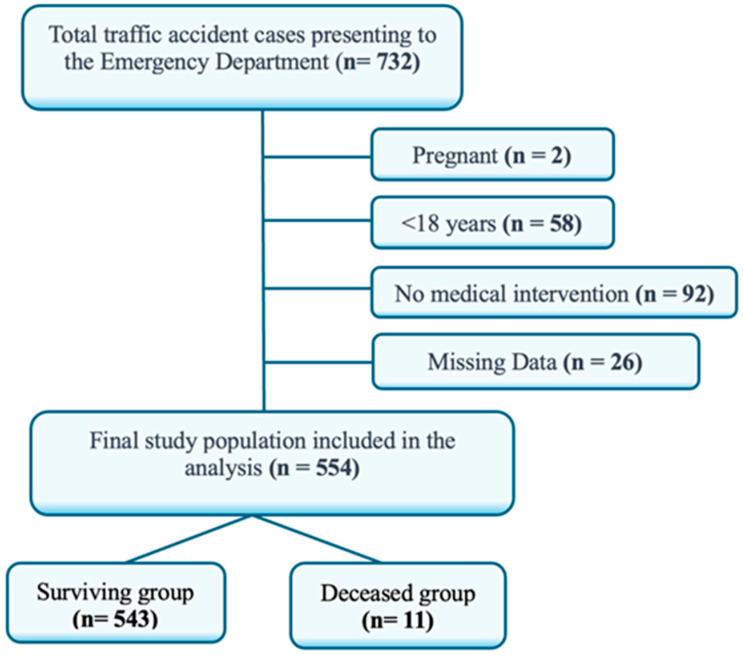
Flow chart of patient selection.

Patients aged 18 years and older who presented to the emergency department with multiple trauma caused by traffic accident-related injuries were included in the study. The exclusion criteria encompassed patients younger than 18 years and those with isolated injuries requiring no medical intervention. Out of 732 patients evaluated after traffic accidents, 178 were excluded: 2 due to pregnancy, 58 because they were under 18 years of age, 92 because they required no medical intervention, and 26 due to incomplete or missing data. A total of 554 patients were enrolled. To ensure a representative sample and minimize selection bias, patients were consecutively recruited during the study period. The patient selection process is summarized in the flow diagram (Figure 1).

### 2.3. Variables and Data Collection

The primary variables of interest included the results of trauma scoring systems (ISS, NISS, AIS, GCS, RTS, TRISS, and APACHE II), mortality outcomes, and patient demographic characteristics (age and gender). Additionally, laboratory parameters such as WBC, hematocrit, bicarbonate, sodium, and potassium were collected to explore potential associations with mortality.

All trauma scores were calculated at the time of the initial presentation. Anatomical scores (ISS, NISS, AIS, and TRISS) were derived using the AIS 15 guide, based on the most severe injuries recorded per patient. Patients were initially evaluated by on-duty emergency medicine residents, and structured case report forms were completed in real time to document clinical and injury-related data. These forms were reviewed the following day by two experienced authors (MK and MT), who independently calculated trauma scores using certified software. All demographic, clinical, and laboratory data were extracted from the hospital’s electronic health records to ensure accuracy and completeness.

### 2.4. Statistical Analysis

Statistical analyses were conducted using SPSS version 27.0 (IBM Corp., Armonk, NY, USA). The normality of the data distribution was assessed using the Shapiro–Wilk test. Quantitative variables were expressed as medians and interquartile ranges (IQRs), while categorical variables were summarized as frequencies and percentages. Group comparisons were performed using the Mann–Whitney U test for non-normally distributed quantitative variables and the chi-square test or Fisher’s exact test for categorical variables. Spearman’s rank correlation analysis was used to assess the relationships between different trauma scoring systems. Receiver Operating Characteristic (ROC) curve analysis was conducted to evaluate the diagnostic performance of each scoring system in predicting mortality. The area under the curve (AUC), sensitivity, specificity, positive and negative likelihood ratios (PLR, NLR), and optimal cutoff values were calculated. A *p*-value < 0.05 was considered statistically significant.

## 3. Results

### 3.1. Demographics of Patients

A total of 554 patients were included in the analysis, comprising 374 males (67.5%) and 180 females (32.5%). The median age of the cohort was 36 years (interquartile range: 25–54 years). Comorbidities were documented in 45 patients (8.1%), with hypertension and diabetes mellitus being the most reported conditions. The most frequently observed trauma types were extremity injuries (61.7%), followed by head and neck trauma (44%), thoracic trauma (29.9%), and abdominal trauma (16.6%). The detailed demographic characteristics, number of comorbidities and trauma-related findings are presented in Table 1.

### 3.2. Laboratory Findings and Trauma Scoring Systems

The comparative analysis revealed significant differences in the laboratory parameters between the deceased and surviving groups. The deceased group had significantly higher median values for the white blood cell (WBC) count and creatinine, and significantly lower values for the hematocrit (Htc) and bicarbonate levels. However, no statistically significant differences were observed between the groups for the sodium and potassium levels (Table 2).

Significant differences were noted in the trauma scoring system results between the two groups. The deceased group had higher median scores for APACHE II (20 vs. 5), AIS (2 vs. 1), ISS (12 vs. 2), NISS (12 vs. 2), and TRISS (0.773 vs. 0.997), and lower scores for RTS (4.09 vs. 7.84) and GCS (4 vs. 15). Detailed comparisons are presented in Table 3.

### 3.3. Correlations Among Trauma Scoring Systems

The correlation analysis demonstrated a perfect positive correlation between the ISS and NISS values (Spearman’s Rho = 0.97, *p* < 0.001). The AIS values also showed very high positive correlations with both the ISS (Spearman’s Rho = 0.88, *p* < 0.001) and NISS. The correlation analysis demonstrated a perfect positive correlation between the ISS and NISS values (Spearman’s Rho = 0.97, *p* < 0.001). The AIS values also showed very high positive correlations with both the ISS (Rho = 0.88, *p* < 0.001) and NISS (Rho = 0.87, *p* < 0.001). Additionally, APACHE II exhibited moderate positive correlations with the ISS (Rho = 0.24, *p* < 0.001), NISS (Rho = 0.23, *p* < 0.001), and AIS (Rho = 0.27, *p* < 0.001). The RTS was negatively correlated with all anatomical scores, particularly the ISS (Rho = −0.32, *p* < 0.001) and NISS (Rho = −0.31, *p* < 0.001). The TRISS score showed strong negative correlations with AIS (Rho = −0.68), ISS (Rho = −0.74), and NISS (Rho = −0.71), and a moderate negative correlation with APACHE II (Rho = −0.52) (all *p* < 0.001). The correlation coefficients for all trauma scoring systems are summarized in Table 4.

**Figure 2:**
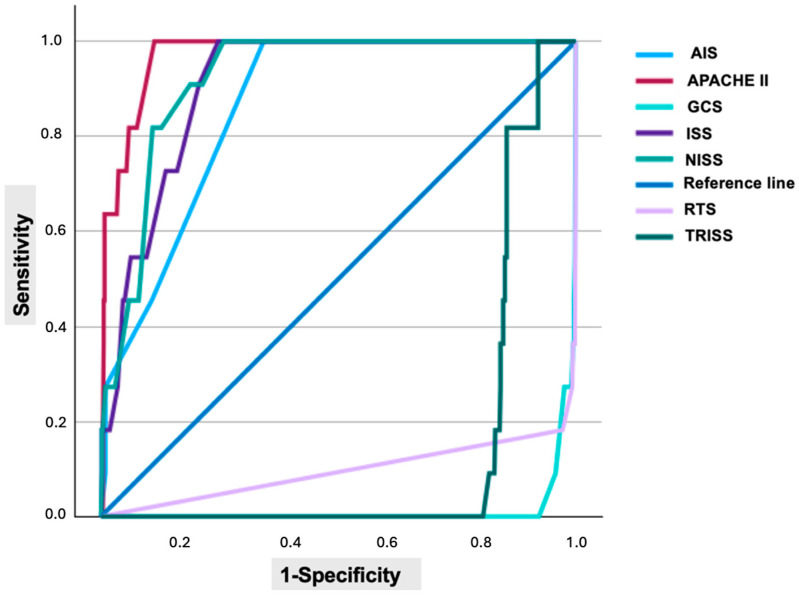
ROC curves of trauma scores for mortality prediction.

The diagnostic performance of trauma scoring systems in predicting mortality was evaluated using ROC curve analysis (Figure 2). All scores demonstrated statistically significant AUC values. The highest AUC was observed for TRISS (0.98), followed by GCS (0.98), APACHE II (0.97), NISS (0.92), ISS (0.91), RTS (0.90), and AIS (0.86). Optimal cutoff values, sensitivity, specificity, likelihood ratios, and AUC values for each scoring system are summarized in Table 5.

## 4. Discussion

Traffic accidents remain a major global cause of trauma-related deaths, accounting for over a million fatalities each year. They are currently the eighth leading cause of death worldwide and may rise to fifth place by 2030 [11]. These figures highlight the importance of early risk assessment and effective management strategies in trauma care.

To support timely decision-making, several trauma scoring systems are commonly used, including ISS, NISS, AIS, RTS, TRISS, and GCS. Combined models like TRISS enhance predictive accuracy by integrating both anatomical and physiological factors. While each system has strengths, they also have limitations. For example, NISS better captures multiple severe injuries in the same region, whereas RTS offers speed but may be less reliable outside hospital settings. These differences underline the need for scoring tools that are both practical and accurate across various clinical settings.

Previous studies have consistently shown that male patients are more frequently involved in traffic-related trauma. For example, Kenarangi et al. [12] reported that 72% of trauma patients were male, with motorcycle accidents being the predominant mechanism. In our study, males also accounted for most cases (67.5%), which is consistent with the greater exposure and risk-taking behavior reported among men. Similarly, the median age in our cohort was 36 years, aligning with global findings that trauma predominantly affects young and middle-aged adults [12,13]. Interestingly, only 8.1% of our patients had comorbid conditions, which is lower than the rates reported in previous studies. This discrepancy may be attributable to regional socioeconomic and cultural factors, as well as differences in patient selection criteria.

Extremity injuries (61.7%) were the most common trauma type in our cohort, followed by head and neck injuries (44%), thoracic injuries (29.9%), and abdominal injuries (16.6%). These findings align with several studies reporting extreme injuries as the most frequent trauma site [14]. However, other studies, such as a 9-year retrospective analysis by Airaksinen, N. K. et al., have identified thoracic and head–neck injuries as the most prevalent [2]. Such differences may reflect variations in vehicle usage, injury mechanisms, and regional patterns of protective equipment use. Moreover, the inclusion of minor injuries in our study, irrespective of severity, may have influenced the frequency distribution of trauma types. Similarly, Kenarangi et al. [12] evaluated nearly 48,000 traffic accident cases and identified motorcycle-related incidents as the leading mechanism of trauma, predominantly affecting male patients. This may also reflect regional patterns in injury mechanisms commonly observed in traffic-related trauma.

When mortality rates in trauma populations are examined, studies conducted in intensive care units or tertiary trauma centers report substantially higher figures. Serviá et al. [15] found a mortality rate of 14% among ICU-admitted trauma patients, while Höke et al. [16] reported an overall rate of 8.2% in a tertiary care setting. In contrast, our study observed a considerably lower mortality rate. This may be attributed to the inclusion of emergency department patients, including those not requiring hospitalization. These findings highlight how patient selection affects reported mortality rates.

### 4.1. Laboratory Parameters and Trauma Scoring Systems

Beyond demographic factors, laboratory markers also play a critical role in trauma prognosis. Several recent studies have emphasized the prognostic significance of laboratory parameters such as hematocrit, creatinine, and bicarbonate in trauma patients. Wu et al. [17] identified these variables as independent predictors of mortality, while Samuthtai et al. [8] noted that the predictive value of hematocrit may vary depending on the timing of measurement and patient characteristics. In line with these findings, our study also demonstrated significant differences in hematocrit, creatinine, and bicarbonate levels between survivors and non-survivors. These markers, which are included in the APACHE II scoring system, appear to contribute meaningfully to early mortality risk stratification. Supporting this, He et al. [18] retrospectively analyzed 569 critically ill trauma patients and identified low pH, elevated lactate and D-dimer, high ISS, and low GCS scores as independent predictors of mortality. Their findings reinforce the prognostic value of both metabolic and clinical parameters, including laboratory indicators and trauma severity.

Combined trauma scoring systems that incorporate both anatomical and physiological parameters have demonstrated strong predictive capabilities. For instance, in a nationwide Korean cohort, Kim et al. [5] found that the TRISS model—which integrates age, sex, RTS, and ISS—had the highest predictive performance among various models, with an AUC of 0.950 and an overall accuracy of 97.1%. Likewise, Ashrafian Fard et al. [10] introduced the TERMINAL-24 score, which effectively predicted both early (<8 h) and in-hospital mortality, with AUC values of 0.964 and 0.954, respectively. Consistent with these findings, our study identified TRISS as the scoring system with the highest AUC (0.98), reinforcing the value of integrated models. The enhanced performance of such approaches likely arises from their capacity to reflect both the extent of anatomical injury and the degree of physiological compromise at presentation.

The prognostic value of APACHE II has been well established, particularly in ICU settings. Kilinc et al. [19] associated scores above 23 with increased mortality, reporting an AUC of 0.83. In our study, APACHE II demonstrated even stronger discriminative power (AUC = 0.97) in a broader emergency department population. However, its reliance on laboratory data—such as creatinine, hematocrit, and bicarbonate—may limit its feasibility during the initial triage phase in emergency settings. These parameters are often unavailable in the prehospital or early ED environment, making rapid calculation of the score difficult. Therefore, despite its high prognostic accuracy, the practical use of APACHE II may be more suitable for in-hospital risk assessment rather than real-time triage decision-making. Similarly, RTS was significantly lower in non-survivors and showed good predictive performance (AUC = 0.90). Although both studies confirmed its utility, our cohort showed more distinct variations in RTS components, especially GCS and respiratory rate, likely reflecting earlier-stage trauma evaluation.

The central role of GCS in this context is further supported by Unger et al. [20], who identified significantly lower GCS scores in non-survivors among polytrauma patients with spinal injuries, emphasizing its prognostic value even in complex trauma presentations. These results support the continued use of RTS and its components, particularly GCS, for early risk stratification in emergency care, and suggest that simplified combinations like ISS + GCS or NISS + RTS may serve as practical alternatives when full laboratory data are not immediately available. Similarly, Ivanova et al. [21] found that in a cohort of severely injured elderly trauma patients (ISS ≥ 16), low GCS scores and severe head injuries were independently associated with 1-year mortality, reinforcing the importance of GCS-based assessment in older populations.

In a recent study by Gupta et al. [22] involving 240 trauma patients, the APACHE II score demonstrated the highest predictive accuracy for mortality (AUC = 0.965), outperforming traditional trauma scores such as RTS, TRISS, and ISS. In their analysis, TRISS and RTS yielded notably low AUCs (0.137 and 0.087, respectively), while ISS followed APACHE II with an AUC of 0.684. By contrast, all scoring systems in our study—including TRISS, APACHE II, GCS, NISS, ISS, and RTS—showed strong predictive performance, with TRISS and GCS achieving perfect sensitivity (100%) and specificity values above 93%. These differences may be due to variations in study populations; Gupta et al. included a high proportion of ICU patients, whereas our cohort consisted solely of emergency department trauma cases. In contrast to their findings, RTS showed reasonable predictive utility in our analysis and may still serve as a useful triage tool given its simplicity and ease of use.

In a prospective study involving elderly trauma patients, Javali et al. [23] demonstrated that all four trauma scoring systems—ISS, NISS, RTS, and TRISS—had excellent predictive value for mortality, with AUCs ranging from 0.947 to 0.972. Similarly, Galvagno et al. [24], in a large multicenter analysis of over 400,000 cases, found that TRISS outperformed both ISS and RTS in predicting trauma-related mortality, highlighting the superior performance of composite scores that integrate anatomical and physiological variables. Consistent with these findings, our study, both ISS and NISS demonstrated strong predictive value for mortality; however, NISS showed a slightly higher AUC (0.92) compared to ISS (0.91). This difference may be attributed to the structural distinction between the two scoring systems. While ISS considers only the most severe injury in each of the three most affected body regions, NISS accounts for the three most severe injuries regardless of location. As a result, NISS can assign a higher score when multiple serious injuries occur within the same anatomical region, potentially offering greater sensitivity in assessing overall injury severity and its prognostic implications. Unlike Javali’s cohort, which focused exclusively on the elderly, our population encompassed a broader age range, suggesting that the discriminative power of these trauma scores remains robust across different demographic groups.

### 4.2. Study Limitations

This study has several limitations. First, although the overall sample size was relatively large, the number of deceased patients was limited. This may affect the precision of some mortality-related analyses, particularly in ROC curve estimations and cutoff point determinations. However, the significant differences observed across multiple scoring systems suggest that the predictive trends remain robust. Second, the single-center design may limit the generalizability of the findings to other settings with different trauma patterns or clinical protocols. Third, minor variability may exist in subjective components such as GCS, despite scores being calculated prospectively by trained researchers. Fourth, the study population consisted solely of adult patients with traffic-related injuries; thus, the findings may not be generalizable to pediatric or geriatric trauma populations with differing physiological responses.

## 5. Conclusions

This study confirms the effectiveness of multiple trauma scoring systems in predicting mortality among emergency department patients with traffic-related multiple trauma. Among these, TRISS and GCS demonstrated the highest discriminative power, followed by APACHE II, NISS, and ISS. While complex scoring models such as TRISS and APACHE II offer greater accuracy, simpler tools like RTS and GCS remain valuable for rapid risk assessment during the early phases of trauma care.

These findings support the integration of both anatomical and physiological scoring systems into routine emergency department evaluation to enhance early risk stratification and guide timely clinical decision making. Further prospective, multicentre studies are needed to validate these results and explore their applicability across different healthcare settings and patient populations.

## Figures and Tables

**Table 1:** Demographics and trauma characteristics of patients.

Demographic Characteristics		
Age (Median (IQR 25–75))		36 (25–54)
Gender *n* (%)	Male *n* (%)	374 (67.5%)
	Female *n* (%)	180 (32.5%)
Number of Comorbidities *n* (%)	No	509 (91.9%)
	1	25 (4.5%)
	2 or more	20 (3.6%)
Trauma Characteristics		
Head and neck trauma		244 (44%)
Thoracic trauma		167 (29.9%)
Trauma of the abdomen		97 (16.6%)
Trauma of the extremities		342 (61.7%)

IQR: Interquartile Range.

**Table 2:** Laboratory values by outcome groups.

Laboratory Parameter	Deceased Group Median (IQR 25–75)	Surviving Group Median (IQR 25–75)	*p*-Value
WBC	15 × 10^3^ (12 × 10^3^–18 × 10^3^)	10 × 10^3^ (8 × 10^3^–12 × 10^3^)	<0.05
Hematocrit (%)	30 (25–35)	40 (35–45)	<0.05
Bicarbonate (mEq/L)	18 (15–20)	22 (20–24)	<0.05
Sodium (mEq/L)	140 (138–142)	141 (139–143)	0.15
Potassium (mEq/L)	4.5 (4.0–5.0)	4.4 (4.2–4.6)	0.20

IQR: Inte IQR: interquartile range, WBC: white blood cell.

**Table 3:** Distribution of trauma scoring system scores by outcome group.

Scoring System	Surviving Group Median (IQR 25–75)	Deceased Group Median (IQR 25–75)	*p* *
APACHE II	5 (4–8)	20 (14.5–25)	<0.001
AIS	1 (1–2)	2 (2–3.5)	<0.001
ISS	2 (1–4)	12 (7.5–15.5)	<0.001
NISS	2 (1–6)	12 (12–22)	<0.001
RTS	7.84 (7.84–7.84)	4.09 (3.46–6.64)	<0.001
GCS	15 (15–15)	4 (3–11)	<0.001
TRISS	0.997 (0.986–0.997)	0.773 (0.451–0.918)	<0.001

* Mann–Whitney U test applied. RTS: Revised Trauma Score; ISS: Injury Severity Score; AIS: Abbreviated Injury Score; NISS: New Injury Severity Score; GCS: Glasgow Coma Scale; APACHE II: Acute Physiology and Chronic Health Evaluation II.

**Table 4:** Correlations between trauma scoring systems.

Scoring System	RTS	AIS	APACHE II	ISS	NISS	GCS	TRISS
RTS	—	Rho: −0.324	Rho: −0.313	Rho:−0.321	Rho: −0.309	Rho: 0.119	Rho: −0.283
		*p* < 0.001	*p* < 0.001	*p* < 0.001	*p* < 0.001	*p* = 0.007	*p* < 0.001
AIS		—	Rho: 0.268	Rho: 0.884	Rho: 0.877	Rho: −0.437	Rho: −0.675
			*p* < 0.001	*p* < 0.001	*p* < 0.001	*p* < 0.001	*p* < 0.001
APACHE II			—	Rho: 0.243	Rho: 0.234	Rho: −0.362	Rho: −0.524
				*p* < 0.001	*p* < 0.001	*p* < 0.001	*p* < 0.001
ISS				—	Rho: 0.971	Rho: −0.430	Rho: −0.741
					*p* < 0.001	*p* < 0.001	*p* < 0.001
NISS					—	Rho: −0.422	Rho: −0.716
						*p* < 0.001	*p* < 0.001
GCS						—	Rho: −0.395
							*p* < 0.001
TRISS							—

Correlation was assessed using Spearman’s rho test.

**Table 5:** Predictive accuracy of trauma scores for mortality based on ROC analysis.

Scoring System	Cutoff Points	Sensitivity 95% CI	Specificity 95% CI	PLR 95% CI	NLR 95% CI	AUC 95% CI	*p*
APACHE II	≥10.5	1 (0.72–1)	0.89 (0.86–0.91)	8.9 (7.0–11.3)	0	0.97 (0.95–0.99)	0.0001
RTS	<7.01	0.82 (0.48–0.98)	0.98 (0.96–0.99)	34.17 (18.6–62.6)	0.19 (0.05–0.67)	0.90 (0.77–0.96)	0.0001
AIS	≥1.50	0.91 (0.59–0.99)	0.66 (0.62–0.70)	2.65 (2.13–3.3)	0.14 (0.02–0.91)	0.86 (0.79–0.93)	0.0001
NISS	≥8.50	0.90 (0.58–0.99)	0.81 (0.77–0.84)	4.79 (3.71–6.18)	0.11 (0.02–0.71)	0.92 (0.88–0.96)	0.0001
ISS	≥4.50	0.82 (0.48–0.98)	0.74 (0.70–0.78)	3.22 (2.35–4.41)	0.24 (0.07–0.84)	0.91 (0.86–0.96)	0.0001
GCS	<9.50	0.73 (0.39–0.94)	0.99 (0.97–0.99)	66.18 (27.6–99.6)	0.28 (0.11–0.73)	0.98 (0.96–0.99)	0.0001
TRISS	<0.972	1 (0.71–1)	0.90 (0.58–0.99)	17.55 (12.5–24.7)	0	0.98 (0.97–0.99)	0.0001

PLR: Positive Likelihood Ratio; NLR: Negative Likelihood Ratio; AUC: Area Under Curve; RTS: Revised Trauma Score; ISS: Injury Severity Score; AIS: Abbreviated Injury Score; NISS: New Injury Severity Score; GCS: Glasgow Coma Scale; APACHE II: Acute Physiology and Chronic Health Evaluation II; TRISS: Trauma and injury severity score.

## Data Availability

The datasets used and/or analyzed during the current study are available from the corresponding author on reasonable request.

## Abbreviations

RTS	Revised Trauma Score
ISS	Injury Severity Score
AIS	Abbreviated Injury Score
NISS	New Injury Severity Score
GCS	Glasgow Coma Scale
TRISS	Trauma and Injury Severity Score
APACHE II	Acute Physiology and Chronic Health Evaluation II
ED	Emergency department
